# Commentary: Clinical efficacy of a Chinese herbal gel plaster combined with manipulation for lumbar disc herniation: a prospective, randomized, double-blind, placebo-controlled trial

**DOI:** 10.3389/fphar.2026.1860420

**Published:** 2026-07-16

**Authors:** Doori Kim, Yoon Jae Lee, In-Hyuk Ha

**Affiliations:** Jaseng Spine and Joint Research Institute, Jaseng Medical Foundation, Seoul, Republic of Korea

**Keywords:** analgesic effect, Haitongpi formula gel plaster, low back pain, lumbar disc herniation, randomized controlled trial

## 1 Introduction

We read with great interest the recent randomized controlled trial (RCT) by Wan et al. (2026), which investigated the efficacy of Haitongpi Formula Gel Paste (HTPGP) in patients with lumbar disc herniation (LDH). The authors successfully demonstrated that integrating a traditional natural product with a modern transdermal drug delivery system can yield significant clinical benefits. However, several observations regarding the underwhelming response of the comparator group and the discrepancy between subjective and objective measures warrant further discussion to ensure a balanced interpretation of the results.

## 2 Underestimation of the manipulation comparator effect

In this study, both groups received “Qing dynasty three-movement manipulation” as a baseline therapy. Manual therapy, such as Chuna or chiropractic manipulation, are a hands-on modality widely practiced across East Asian integrative medicine (Kim et al., 2023), with an established evidence base for LDH (Mo et al., 2018; Santilli et al., 2006). Notably, prior RCTs comparing chiropractic manipulation to sham interventions reported substantial visual analog scale (VAS) reductions, often exceeding 3 points from a baseline of approximately 6.5 (Santilli et al., 2006). Of course, direct comparison of effect sizes is limited by differences between the two patient populations. For example, although both studies enrolled patients with MRI-confirmed disc herniation and radiating leg pain, Wan et al. excluded patients with severe pain (VAS >7). Nonetheless, multiple studies consistently indicate that manipulation-based interventions produce clinically meaningful pain reductions in LDH. A network meta-analysis of 121 RCTs including over 13,000 patients with LDH further confirmed the superiority of Tuina and acupuncture over traction and herbal medicine (Mo et al., 2019).

Considering this evidence base, the response observed in the comparator arm of Wan et al. is strikingly modest. After 2 weeks of treatment, VAS of the comparator group (manipulation + Placebo Plaster) declined only from 5.80 to 4.13 — a within-group change of 1.67 points. Reported minimal clinically important difference (MCID) values for low back pain and radiating leg pain vary across studies, ranging from approximately 1.5–3.5 points (Solberg et al., 2013; Copay et al., 2008). However, 2.0 points remains the most widely accepted threshold. (Childs et al., 2005; Ostelo et al., 2008; Ostelo and de Vet, 2005). The within-group change in the comparator group of this trial fell below this MCID. Furthermore, scores drifted upward after treatment cessation, reaching 4.49 at day 42, indicating that comparator participants remained in the moderate-pain range throughout follow-up. This outcome is discordant with the evidence base cited by the authors themselves, including Mo et al. (2018), which they invoke as justification for the comparator choice.

This raises questions regarding the Qing manipulation protocol. The lack of detailed procedural descriptions or standardized intensity metrics makes it difficult to determine if the manual therapy was administered at a therapeutic threshold. If the baseline manipulation was suboptimal, the “add-on” effect of HTPGP might be statistically exaggerated. Readers should cautiously consider whether the significant intergroup difference reflects the superior potency of HTPGP or the relative inefficacy of the specific manipulation protocol used as a comparator.

## 3 Clinical significance of intergroup differences

While the HTPGP group achieved an MCID-level improvement, the difference between the two groups remained relatively narrow. Clinical significance is often judged by whether an intervention provides a perceptible benefit over the current standard of care. Given that the comparator group’s performance was lower than expected for standard manual therapy, it remains unclear if the HTPGP’s advantage would hold when compared to a more robust, active physical therapy regimen. Furthermore, the statistical significance reported may not fully translate into a “perceptible shift” in the patient’s quality of life beyond the immediate analgesic sensation.

## 4 Mechanistic implications: sEMG and post-treatment exacerbation

A critical point of discussion is the lack of significant findings in surface electromyography (sEMG). While VAS and Oswestry Disability Index (ODI) scores improved, the objective physiological markers remained unchanged at day 14. One possible interpretation is that HTPGP’s mechanism is primarily centered on peripheral analgesia and the modulation of local inflammatory nociceptors rather than structural disease modification or functional neuromuscular restoration. Of course, this interpretation should be regarded as one of several possibilities. The null sEMG findings may equally reflect intrinsic limitations of the measurement approach used in this trial: surface electrodes have reduced sensitivity for deeper paraspinal musculature such as the multifidus (Stokes et al., 2003); the 14-day assessment window may be insufficient to capture neuromuscular adaptation, which typically follows pain resolution with a delay (Hodges and Tucker, 2011); and the analysis was restricted to the multifidus and erector spinae, leaving other contributors to lumbar stability (e.g., transversus abdominis, quadratus lumborum) unexamined. Trials with extended follow-up, broader muscle sampling, and complementary imaging or finer-grained electrophysiological techniques are required.

## 5 Interpreting the adverse event pattern in the comparator arm

A notable design feature of this study is the apparent physical equivalence of the placebo and active patches, evidenced by a higher incidence of mild skin reactions in the placebo arm. This pattern is consistent with adequate blinding at the level of cutaneous sensation. However, the reverse interpretation cannot be excluded: the perception of skin stimulation without accompanying analgesic relief in the placebo arm may have contributed to partial unblinding and a nocebo response. It could have further attenuated the comparator arm’s improvement. The available data do not allow these two possibilities to be distinguished. Future trials should therefore prioritize a more carefully matched comparator patch that closely approximates HTPGP in both physical and sensory properties. This would help minimize differential expectancy effects between groups.

## 6 Concluding remarks

In conclusion, Wan et al. provide valuable clinical evidence supporting HTPGP as a safe and effective adjunct for short-term pain relief in patients with LDH. However, the underwhelming performance of the comparator group and the interpretive issues outlined above suggest that the relative clinical benefit of HTPGP should be interpreted with caution.

To address these limitations, we propose three directions for future research: (1) standardized reporting of the manual therapy protocol using the TIDieR checklist; (2) extension of follow-up to at least 6 months; and (3) serial sEMG assessment with finer-grained techniques such as fine-wire EMG or musculoskeletal ultrasound. Such methodological refinements will allow the true incremental benefit of HTPGP to be more confidently determined.
